# The association between pain catastrophizing, physical function and pain in a cohort of patients undergoing knee arthroplasty

**DOI:** 10.1186/s12891-019-2787-6

**Published:** 2019-09-12

**Authors:** Sara Birch, Maiken Stilling, Inger Mechlenburg, Torben Bæk Hansen

**Affiliations:** 1 Department of Physiotherapy and Occupational Therapy, Holstebro Regional Hospital, Hospital Unit West, Holstebro, Denmark; 2 Department of Clinical Medicine, Aarhus University, Aarhus, Denmark; 3 University Clinic for Hand, Hip, and Knee surgery, Holstebro Regional Hospital, Hospital Unit West, Holstebro, Denmark; 4 Department of Orthopaedic Surgery, Aarhus University Hospital, Aarhus, Denmark; 5 Department of Public Health, Aarhus University, Aarhus, Denmark

**Keywords:** Knee arthroplasty, Pain catastrophizing, Physical function, Pain

## Abstract

**Background:**

Pain catastrophizing contributes to acute and long-term pain after knee arthroplasty (KA), but the association between pain catastrophizing and physical function is not clear.

We examined the association between preoperative pain catastrophizing and physical function one year after surgery, as well as differences in physical function, pain and general health in two groups of patients with high and low preoperative pain catastrophizing score.

**Methods:**

We included 615 patients scheduled for KA between March 2011 and December 2013. Patients completed The Pain Catastrophizing Scale (PCS) prior to surgery. The Oxford Knee Score (OKS), Short Form-36 (SF-36) and the EuroQol-5D (EQ-5D) were completed prior to surgery, and 4 and 12 months after the surgery.

**Results:**

Of the 615 patients, 442 underwent total knee arthroplasty (TKA) and 173 unicompartmental knee arthroplasty (UKA). Mean age was 67.3 (SD: 9.7) and 53.2% were females. Patients with PCS > 21 had statistically significantly larger improvement in mean OKS for both TKA and UKA than patients with PCS < 11; 3.2 (95% CI: 1.0, 5.4) and 5.4 (95% CI: 2.2, 8.6), respectively. Furthermore, patients with preoperative PCS > 21 had statistically significantly lower OKS, SF-36 and EQ-5D and higher pain score than patients with PCS < 11 both preoperatively and 4 and 12 months postoperatively.

**Conclusions:**

Patients with high levels of preoperative pain catastrophizing have lower physical function, more pain and poorer general health both before and after KA than patients without elevated pain catastrophizing.

**Electronic supplementary material:**

The online version of this article (10.1186/s12891-019-2787-6) contains supplementary material, which is available to authorized users.

## Introduction

For persons with end-stage knee osteoarthritis, knee arthroplasty (KA) is common treatment for pain and disability when non-surgical management is no longer effective. Improvement in function and durability of total knee replacement (TKA) procedures has been documented by several researchers [1–3]. Although the procedure is safe and highly successful, patients’ satisfaction rate following TKA is only around 80% [4]. This has led several investigators to evaluate patients undergoing KA to try to determine preoperative factors that might contribute to better or worse outcomes.

Several risk factors for poor outcome after KA are found. These factors can be divided into physical and psychological predictors. The former include young age, female gender, obesity, severe preoperative knee pain and other painful joints [5–8]. The latter include depression, anxiety and pain catastrophizing [6, 9]. From a clinical perspective, research in these psychological factors is important because it helps us to identify factors warranting our attention when designing interventions to improve outcome after KA.

Studies have found pain catastrophizing to be a consistent psychologic predictor of persistent pain six months to two years after TKA [10–12], while others have failed to replicate this result [9, 13]. Recently, a systematic review stated that only few studies have followed patients more than three months after TKA [9].

Pain catastrophizing is characterized as negative emotional and cognitive responses to actual or anticipated pain. It is often described as a set of maladaptive beliefs and consist of several components such as rumination, helplessness in coping with pain, excessive worry and exaggerated attention against pain-related thoughts [14]. Furthermore, pain catastrophizing is hypothesized to impact health behaviours such as physical activity, and the fear avoidance model describes how catastrophic thoughts about pain might result in further pain-related fear, avoidance and disability [15]. Because pain catastrophizing and fear avoidance can be related to these negative patient outcomes, clinicians need to be aware of these behaviours and research suggest that cognitive behavioural therapy is associated with significant reductions in pain catastrophizing [16, 17].

Despite the hypothesized association between pain catastrophizing and disability we only identified two studies that directly assessed the role of pain catastrophizing on physical function after KA [11, 12]. The primary aim of this study was to analyse the association between preoperative pain catastrophizing and postoperative self-reported function measured with the Oxford Knee Score (OKS). Secondly, we wanted to investigate possible differences in self-reported physical function, pain and general health among two groups of patients with high and low preoperative pain catastrophizing score. We hypothesized that pain catastrophizing negatively affects/impacts patients’ function, pain and general health during the first year after surgery.

## Methods

### Study population

The study is a prospective observational cohort study. All patients were recruited between March 2011 and December 2013. Eligibility criteria included being listed for a primary unicompartmental knee arthroplasty (UKA) or a TKA. Patients who did not speak or read Danish or did not attend the preoperative education day were not included. If a patient had undergone primary TKA or UKA in the contralateral limb during the study period, the patient only participated in the study with data from the first KA to avoid multiple observations on some patients. All surgeries were performed by 4 highly experienced knee arthroplasty surgeons.

The patients completed the 4 questionnaires: Pain Catastrophizing Scale (PCS), Oxford Knee Score (OKS), The Physical Function domain of Short Form-36 (SF-36 (PF)) and EuroQol 5D (EQ-5D) at the time of their preoperative education day approximately one week before KA. Furthermore, they completed OKS, SF-36 (PF) and EQ-5D at the time of their postoperative follow-up, 4 and 12 months after KA. Additional preoperative variables collected from the Lundbeck Foundation Centre for Fast-Track Hip and Knee replacement database (LCDB) to control for confounding were: age, gender, body mass index (BMI), alcohol consumption, smoking, living alone and co-morbidity (cardiac disease, pulmonary disease, high blood pressure, hypercholesterolemia, diabetes, psychiatric disorder, previous stroke and previous venous thromboembolic event).

### The pain catastrophizing scale

The Pain Catastrophizing Scale (PCS) consists of 13 questions addressing feelings and thoughts related to the experience of pain (see Additional file 1). Sullivan et al. [18] developed the scale in 1995, and it was later validated and translated into Danish. The Danish version is considered valid for use in both clinical and non-clinical samples, and the internal consistency is found acceptable [19]. Each question is answered on a 5-point Likert scale with 0 being “not at all” and 4 being “all the time”, giving a total score ranging from 0 to 52. The higher the score, the more catastrophizing thoughts are present. The PCS consists of three subscales/dimensions of catastrophizing: rumination, magnification and helplessness [18].

In this study, the secondary aim was to investigate possible differences in pain, general health and physical function among two groups of patients with high and low preoperative PCS. We used the 33rd and the 66th percentile to split the patients, meaning that patients with PCS < 11 were defined as “non-catastrophizers” and patients with PCS > 21 were defined as “catastrophizers”.

### Questionnaires

The OKS is a joint-specific questionnaire consisting of 12 questions covering function and pain associated with the knee. Each item is scored from 0 to 4. Overall scores run from 0 to 48 with 48 being the best outcome [20].

Pain was measured from question 1 in the OKS; “How would you describe the pain you usually have in your knee?” The question is answered on a 5-point Likert scale ranging from 0 indicating severe pain to 5 indicating no pain [20].

The SF-36 is a widely used generic measure consisting of 36 questions in eight different domains. In this study, we used only one domain, physical function (PF). The PF domain consists of 10 questions and is scored on a scale from 0 to 100, with 100 indicating no problems [21].

The EQ-5D is a standardised generic measure of self-reported general health and consists of 5 dimensions: mobility, self-care, usual activities, pain/discomfort and anxiety/depression [22].

### Statistical analysis

Statistical analysis was performed using STATA 15. We used visual QQ-plots to determine if data were normally distributed or not and descriptive statistics to summarise patient characteristics and baseline data. Categorical data are presented as number and percentage, and continuous data are presented as mean and standard deviation (SD), if normally distributed, and median and interquartile range (IQR), if not normally distributed. The PCS was divided into three groups with cut-off at PCS < 11 and PCS > 21. Numbers of co-morbidities were summed and dichotomized 0 or ≥ 1. Pain was dichotomized in “no pain” (none/very mild/mild) and “pain” (moderate/severe). Missing values were filled in with mean values as described in the manuals if less than half of the answers were missing in the SF-36 (PF) [21] and if two or fewer of the answers were missing in the OKS and the PCS [18, 23].

We used multiple linear regression to determine the association between preoperative PCS and change in OKS score from pre- to 12 months postoperatively. The baseline characteristics presented in Table 1 (sex, age, BMI, alcohol, smoking, operated bilateral and co-morbidity) were considered as potential covariates and adjusted for in regression analyses. A *p*-value of < 0.05 was considered significant for all statistical tests. 95% confidence interval was defined as (95% CI).

**Table 1:** Patient demographics and characteristics

	Study population *n* = 615	TKA *n* = 442	UKA *n* = 173
Female (%)	320 (52.0)	240 (54.3)	80 (46.3)
Age (SD)	67.3 (9.7)	67.9 (9.5)	65.6 (9.9)
BMI^a^ (SD)	29.0 (4.5)	29.1 (4.5)	28.9 (4.2)
Alcohol > 24 g/day^a^	36 (6.3)	26 (6.2)	10 (6.2)
Smoking (%)^a^	82 (14.5)	64 (15.3)	18 (11.1)
Co-morbidity (%)^a^	436 (72.2)	320 (73.7)	116 (69.0)
Operated bilateral (%)^c^	61 (9.9)	42 (9.5)	19 (11.0)
SF-36 (PF)^a^(SD)	19.0 (4.1)	18.5 (4.1)	20.1 (3.8)
EQ 5D (IQR)^b^	0.723 (0.144)	0.723 (0.129)	0.723 (0.121)
OKS (SD)	25 (6.3)	24.4 (6.4)	26.6 (6.7)
PCS (IQR)	16 (17)	17 (17)	13 (14)

Co-morbidity is defined as ≥1 diseases

^a^Missing data on 11 patients

^b^Missing data on 53 patients.

^c^Patients having two knee arthroplasties within 12 months

To test the association between preoperative PCS and pain after 12 months, we used logistic regression.

Differences in the OKS and the SF-36 between patients with high and low pain catastrophizing score were measured using linear mixed effects model with a random person level and systematic effects of BMI, time, group and the interaction between time and group. Model validation was performed by comparing observed and expected within-subject standard deviations and correlations and by inspecting QQ-plots.

Differences in pain and the EQ-5D between patients with high and low pain catastrophizing score were measured using chi square test and Wilcoxon signed-rank test.

## Results

**Fig. 1:**
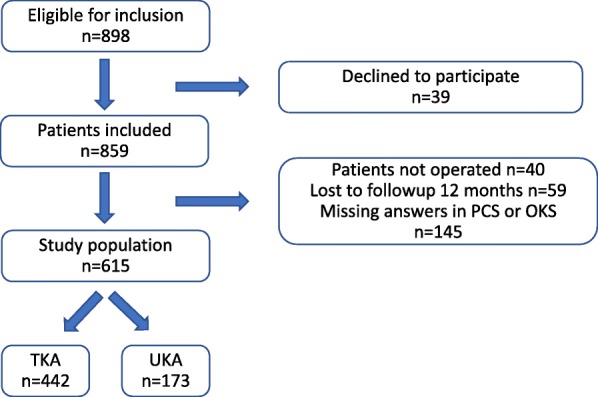
Flow chart

Figure 1 shows the flow of patients through the study. From March 2011 to December 2013, 898 patients attended the preoperative educational day and were available for recruitment. Of these, 39 declined to participate and 859 patients were included. We excluded 244 patients; 40 were not operated, 59 were lost to follow-up at 12 months, and 145 did not completely fill-out either the PCS preoperative or the OKS pre- or postoperatively. The final study population consisted of 615 patients, of whom 61 patients had two knee arthroplasties within 12 months. We found no statistically significantly differences for age (*p* = 0.15) and gender (*p =* 0.07) between the patients excluded and the patients in the final study group. Although there was a trend indicating that the female ratio is lower in the final study group than in the patients excluded. Furthermore, we found no differences between the two groups in PCS (*p* = 0.61) or OKS (*p* = 0.16) (These analyses only included the patients who answered the questionnaire).

The patient characteristics at baseline are presented in Table 1. The study population consisted of 615 patients, 52% female, with a mean age of 67.3 years. A total of 442 of the patients had TKA and 173 UKA. The preoperative median PCS score was 13 (IQR 14) among UKA patients and 17 (IQR 17) among TKA patients.

Preoperative pain catastrophizing was associated with the change in the OKS 12 months after the operation (Table 2). “Catastrophizers” had statistically significantly larger improvements in mean OKS for both TKA and UKA than “non-catastrophizers”; 3.2 (95% CI: 1.0, 5.4) and 5.4 (95% CI: 2.2, 8.6) respectively.

**Table 2:** Association between preoperative pain catastrophizing score and change in Oxford Knee Score (OKS) from preoperatively to 12 months postoperatively

	OKS total score		
Variable	Change in OKS ^a^	(95% CI)	*P* value
TKA (*n* = 419)			
PCS < 11	ref		
PCS =11–21	0.9	(−1.5, 3.2)	0.47
PCS > 21	3.2	(1.0, 5.4)	0.005
UKA (*n* = 162)			
PCS < 11	ref		
PCS = 11–21	4.3	(1.5, 7.1)	0.003
PCS > 21	5.4	(2.2, 8.6)	0.001

^a^Adjusted for sex, age, BMI, alcohol, smoking, operated bilateral (patients having two arthroplasties within 12 months) and co-morbidity

Table 3 shows the association between preoperative PCS and pain measured 12 months after KA. The odds ratio (OR) indicates that “catastrophizers” have a 2.7 (95% CI: 1.4, 5.2) higher odds of getting moderate to severe pain 12 months after TKA than “non-catastrophizers”. For UKA, the same pattern applied: OR 4.8 (95% CI: 1.1, 21.7); but the 95% CIs are wide and the association not as strong.

**Table 3:** Association between preoperative pain catastrophizing score and pain measured 12 months after knee arthroplasty

	Moderate/severe pain vs mild/very mild/no pain		
Variable	Odds ratio^a^	(95% CI)	*P*-value
TKA (n = 419)			
PCS < 11	ref		
PCS 11–21	2.2	(1.1, 4.4)	0.03
PCS > 21	2.7	(1.4, 5.2)	0.003
UKA (*n* = 159)			
PCS < 11	ref		
PCS 11–21	0.4	(0.1, 2.8)	0.44
PCS > 21	4.8	(1.1, 21.7)	0.04

^a^Adjusted for sex, age, BMI, alcohol, smoking, operated bilateral (patients having two arthroplasties within 12 months) and co-morbidity

Table 4 describes differences in patient demographic, characteristics and self-reported outcome preoperatively and 4 and 12 months postoperatively. There were no differences in patient characteristics and demographics among “catastrophizers” and “non-catastrophizers” except for “catastrophizers” having higher BMI, and more using a walking aid.

**Table 4:** Differences in patient characteristics and self-reported outcome between patients with high and low pain catastrophizing score

	All patients			TKA		UKA	
	PCS < 11	PCS > 21	*P*-value	PCS < 11	PCS > 21	PCS < 11	PCS > 21
	*n* = 205	*n* = 207		*n* = 138	*n* = 165	*n* = 67	*n* = 42
Female (%)	106 (51.7)	112 (54.1)	0.63	73 (52.9)	92 (55.8)	33 (49.3)	20 (47.6)
Age years (SD)	67.7 (9.2)	66.7 (10.0)	0.37	68.1 (9.0)	67.0 (9.9)	66.7 (9.5)	65.9 (10.4)
BMI kg/m^2^ (SD)	28.2 (4.3)	29.7 (4.4)	< 0.001	28.0 (4.2)	29.8 (4.5)	28.7 (4.4)	29.3 (4.1)
Alcohol > 24 g/Day (%)	11 (5.6)	9 (4.6)	0.70	7 (5.2)	8 (5.1)	4 (6.5)	1 (2.3)
Smoking (%)	25 (12.6)	33 (16.9)	0.22	21 (15.4)	27 (17.2)	4 (6.5)	6 (15.8)
Co-morbidity^a^ (%)	139 (67.8)	153 (73.9)	0.24	100 (72.5)	124 (75.2)	39 (58.2)	29 (69.0)
Walking aid (%)	24 (12.1)	38 (19.5)	0.03	21 (15.4)	30 (19.1)	3 (4.8)	8 (21.1)
Living alone (%)	53 (26.8)	42 (21.5)	0.23	37 (27.2)	33 (21.7)	16 (25.8)	9 (23.7)
Operated bilateral^b^ (%)	20 (9.7)	16 (7.7)	0.52	13 (9.4)	13 (7.9)	7 (10.5)	7 (7.1)
Outcome							
Preoperatively							
OKS (IQR) (*n* = 412)	29 (9)	21 (8)	< 0.001	28 (10)	21 (8)	31 (8)	22 (8)
Pain (%) (n = 412)	127 (61.2)	187 (90.3)	< 0.001	89 (64.5)	152 (92.1)	38 (56.7)	35 (83.3)
SF-36 (PF) (IQR) (*n* = 404)	50 (35)	35 (25)	< 0.001	50 (35)	35 (25)	57.5 (30)	35 (20)
EQ 5D (IQR) (*n* = 376)	0.72 (0.08)	0.66 (0.33)	< 0.001	0.72 (0.05)	0.66 (0.33)	0.77 (0.10)	0.59 (0.40)
4 months							
OKS (IQR) (*n* = 336)	39 (10)	36 (12)	0.001	37 (11)	35 (11)	41 (8)	37 (12)
Pain (%) (n = 412)	32 (17.7)	56 (33.9)	0.001	25 (20.8)	47 (36.2)	7 (11.5)	9 (25.7)
SF-36 (PF) (IQR) (*n* = 346)	80 (25)	75 (30)	0.004	75 (30)	75 (30)	85 (15)	80 (30)
EQ 5D (IQR) (*n* = 331)	0.82 (0.23)	0.82 (0.28)	0.023	0.82 (0.28)	0.82 (0.28)	0.84 (0.18)	0.82 (0.23)
12 months							
OKS (IQR) (n = 412)	42 (10)	38 (13)	< 0.001	41 (11)	37 (14)	44 (8)	41 (8)
Pain (%) (n = 412)	21 (10.2)	49 (23.7)	< 0.001	16 (11.6)	42 (25.5)	5 (7.5)	7 (16.7)
SF-36 (PF) (IQR) (*n* = 401)	85 (25)	75 (30)	0.001	80 (30)	75 (32.5)	85 (20)	80 (20)
EQ 5D (IQR) (*n* = 397)	0.84 (0.22)	0.82 (0.28)	0.002	0.84 (0.22)	0.82 (0.28)	1 (0.28)	0.82 (0.23)

Parentheses are percentage unless otherwise specified. Pain is dichotomized from OKS q1 in “no pain” (none/very mild/mild) and “pain” (moderate/severe). *P*-value for outcome is measured from multivariate repeated measurements ANOVA for OKS and SF36 (PF) and chi square test and Wilcoxon signed-rank test for pain and EQ.5D

^a^cardiac disease, pulmonary disease, high blood pressure, hypercholesterolaemia, diabetes, psychiatric disorder, previous stroke, and previous venous thromboembolic event

^b^Patients having two knee arthroplasties within 12 months

**Fig. 2:**
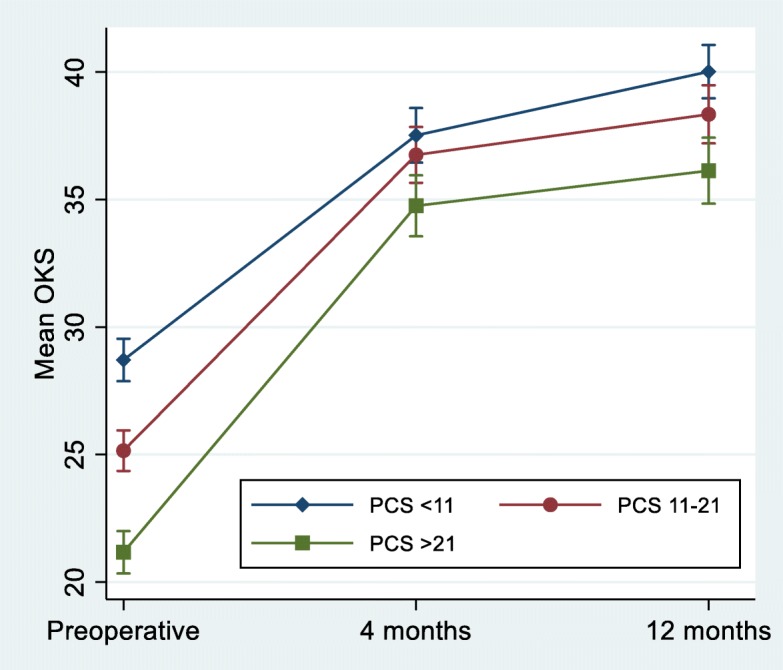
Mean Oxford Knee score for patients with low (*n* = 205), moderate (*n* = 203) and high (*n* = 207) preoperative Pain Catastrophizing Score. Error bars represent 95% confidence intervals

“Catastrophizers” have 7.5 (95% CI: 6.4, 8.7) point lower OKS score preoperative and 3.9 (95% CI: 2.2, 5.5) point lower score after 12 months than “non-catastrophizers” (Fig. 2 and Table 5). The same is applied for the SF-36 (PF) (Table 5) and the EQ-5D (Table 4). Furthermore, a statistically significantly higher number of the “catastrophizers” had moderate/severe pain both preoperatively and 4 and 12 months postoperatively.

**Table 5:** Differences in mean Oxford Knee Score and mean SF-36 (PF) for patients with low and high preoperative Pain Catastrophizing Score

	Low (n = 205) vs high (n = 207) PCS					
Time	OKS	95% CI	*P* value	SF36 (PF)	95% CI	*P* value
Preoperative	7.5	(6.4, 8.7)	0.000	16.7	(12.7, 20.6)	0.000
4 months	2.8	(1.2, 4.3)	0.001	6.2	(2.0, 10.5)	0.004
12 months	3.9	(2.2, 5.5)	0.000	7.0	(2.4, 11.7)	0.003

Both patients with TKA and UKA experienced the largest improvements from preoperatively to 4 months postoperatively with smaller improvements thereafter (tested with a repeated measure ANOVA with time as a factor *p* < 0.001). Patients with UKA reported better function in the OKS and the SF-36 (PF) and less proportion of patients reported moderate/severe pain than the TKA patients. This applied to both “catastrophizers” and “non-catastrophizers”.

## Discussion

The primary aim of this study was to analyse the association between preoperative pain catastrophizing and postoperative function measured with the OKS 12 months after the operation.

We found that preoperative PCS was associated with changes in the OKS from preoperatively to 12 months postoperatively. “Catastrophizers” had larger improvements in OKS than “non-catastrophizers”, yet, they reported significantly lower OKS, SF36 (PF) and EQ. 5D scores and more pain both preoperatively and 4 and 12 months postoperatively.

### PCS and physical function

The current evidence regarding the potential impact of PCS on physical function is conflicting. Sullivan et al. found that pain catastrophizing predicted both pain and function 12 months after TKA [12]. Similarly, Bierke et al. found that patients with high PCS had a significantly lower total KOOS and a higher pain score preoperatively and 6 months postoperatively. However, they were not able to find this association 12 months postoperatively [24]. Riddle et al. followed 140 patients and found that a PCS score of 16 or higher predicted pain outcome after KA but not function [11]. None of these studies investigated change in scores from before to after surgery.

Contrary to our expectation, we found that “catastrophizers” reported significantly larger improvements in mean OKS TKA; 3.2 (95% CI: 1.0, 5.4) than “non-catastrophizers”, possibly because their preoperative score on the OKS scale was lower. We know that the expected score change depends on the preoperative score, and that patients with lower preoperative physical function normally improve more than patients with higher physical function [23, 25].

Based on the patients’ preoperative OKS score, Murray et al. reported from the Knee Arthroplasty Trial mean OKS data before and after TKA divided into 10 subgroups based on OKS score and our results are similar indicating that the difference in preoperative score may be one of the reasons for the larger improvement among “catastrophizers” [23]. Whether PCS has a predictive value for physical function after KA or whether the larger improvement in OKS score among “catastrophizers” than among “non-catastrophizers” can be explained by the lower preoperative score before KA needs to be further investigated.

Furthermore, a difference of 5 points in the OKS between the two groups is a minimal important difference in change score from baseline [26]. The mean difference in change scores between “catastrophizers” and “non-catastrophizers” in this study was 3.2 (95% CI: 1.0, 5.4) for TKA and 5.4 (95% CI: 2.2, 8.6) for UKA. So, even though the differences are statistically significant, only the results from UKA are clinically relevant.

### PCS and pain

Lazaradoi et al. followed 121 patients with knee osteoarthritis in a period of seven days and found that daily physical activity was associated with higher levels of knee pain among patients with high PCS than among patients with low PCS [27]. In the present study, we found that “catastrophizers” had 170 (95% CI: 40, 420)% higher odds of reporting moderate to severe pain 12 months after a TKA than “non-catastrophizers”. Like in our study, Riddle et al. found that patients with PCS > 16 more often experienced improvements below 50% on the WOMAC pain scale (OR: 2.67; 95% CI: 1.2, 6.1) [11], and Forsythe el al. found that patients with high preoperative PCS were more likely to experience persistent pain and disability up to two years after their operation [10]. Our findings add to the current evidence suggesting that pain catastrophizing has a negative influence on the intensity and duration of the pain experience [9, 28]. Despite the fact that pain catastrophizing may be a predictor of persistent pain after TKA currently there are only limited treatment options and a recent study by Riddle et al. shows that cognitive behaviorally based pain coping skills training for patients with moderate to high pain catastrophizing does not seem to improve pain or disability outcomes after TKA [29].

### PCS cut-off scores

In the present study we decided to split the patients into three groups of equal size based on their PCS score. The reason for this is that we wanted to study the patients in subgroups based on evidence saying that patients with higher scores have poorer outcomes and that up to one third of the patients report poor outcome. We are aware that our cut-points are based on a statistical dichotomization and that the PCS manual define patients with a PCS > 30 to be at high risk of developing chronic pain, but only 91 of the patients in this study reported a PCS > 30 and this gives us too few patients in the high group too divide the patients into TKA and UKA. Additional analysis made with cut-off scores at 21 and 30 as recommended by the manual did not change the results (data not shown, see Additional file 2). Only limited research is available on pain catastrophizing cut-off scores indicating that more research in this area is needed.

### Strengths and limitations

One of the strengths of this study is its large number of patients compared with other similar studies [11, 12]. Furthermore, we followed the patients for 12 months, which we consider a strength since a recent systematic review has pointed out that only few studies have followed the patients for more than three months [9]. There are however some limitations. First, a relatively large number (31.8%) of the patients did not enter the analysis. These patients were evenly distributed over PCS groups and we found no differences in age and gender. Hence, this is unlikely to have biased the results in the direction of any particular group of patients. Second, at 4 months of follow-up, approximately 18% did not answer the questionnaires, and the results at this time point are not as certain as the preoperative and 12-month results. However, the primary end-point in this study was 12 months after the operation so this has no consequence for the primary results.

## Conclusions

Despite these limitations, our study shows that preoperative catastrophic thinking in relation to pain may be a risk factor for postoperative pain 12 months after KA. Furthermore, our results shows that there is a statistically significantly difference between “catastrophizers” and “non-catastrophizers” in physical function and quality of life both preoperative and 4 and 12 months postoperative. However, this difference is small and 12 months postoperative it is only clinically relevant for the patients operated with UKA and not TKA. PCS scores are not used routinely as screening before KA, but PCS scores may be important to the surgeon in advising the patient about the results of KA surgery and more research is needed to determine the association with pain and physical function and define precise cut-off points. Formerly, pain catastrophizing was considered a stable factor over time, but recent research challenges this evidence [13], so interventions designed to reduce pain catastrophizing may have the potential to improve pain outcome and physical function in “catastrophizers” after KA.

## Additional files

Additional file 1: Pain catastrophizing scale. The questionnaire: Pain catastrophizing scale. (PDF 193 kb)

Additional file 2: Additional analysis. Analysis with PCS cut-off score at 21 and 30. (DOCX 16 kb)

## Data Availability

All data used and analysed during the current study are available from the corresponding author on reasonable request.

## Abbreviations

95% CI: 95% confidence interval
BMI: Body mass index
EQ-5D: EuroQol-5D
IQR: Interquartile range
KA: Knee arthroplasty
OKS: Oxford Knee Score
PCS: Pain Catastrophizing Scale
SD: Standard deviation
SF-36: Short Form-36
TKA: Total knee arthroplasty
UKA: Unicompartmental knee arthroplasty
