# Supplementary material for: The association between pain catastrophizing, physical function and pain in a cohort of patients undergoing knee arthroplasty
Source: BMC Musculoskelet Disord. 2019 Sep 12;20:421. doi: 10.1186/s12891-019-2787-6 (PMC6739909; doi:10.1186/s12891-019-2787-6)
Supplement: Supplementary file 1 — Pain catastrophizing scale. The questionnaire: Pain catastrophizing scale. (PDF 193 kb) [file 12891_2019_2787_MOESM1_ESM.pdf]

Pain Catastrophizing Scale

Sullivan MJL, **Bishop** S, **Pivik** J. (1995)

Name: Age: Gender: Date:

------------------------------ ---------- ☐Male ☐Female ----------------------

Everyone experiences painful situations at some point in their lives. Such experiences may include headaches, tooth pain, joint or muscle pain. People are often exposed to situations that may cause pain such as illness, injury, dental procedures or surgery.

Instructions:
We are interested in the types of thoughts and feelings that you have when you are in pain. Listed below are thirteen statements describing different thoughts and feelings that may be associated with pain. Using the following scale, please indicate the degree to which you have these thoughts and feelingswhen you are experiencing pain.

RATING	0	1	2	3	4
MEANING	Not at all	To a slight degree	To a moderate degree	To a great degree	All the time

When I'm in pain …

Number	Statement	Rating
1	I worry all the time about whether the pain will end.	
2	I feel I can't go on.	
3	It's terrible and I think it's never going to get any better	
4	It's awful and I feel that it overwhelms me.	
5	I feel I can't stand it anymore	
6	I become afraid that the pain will get worse.	
7	I keep thinking of other painful events	
8	I anxiously want the pain to go away	
9	I can't seem to keep it our of my mind	
10	I keep thinking about how much it hurts.	
11	I keep thinking about how badly I want the pain to stop	
12	There's nothing I can do to reduce the intensity of the pain	
13	I wonder whether something serious may happen.	

Copyright 1995 Michael J.L. Sullivan. Reproduced with permission.
Source: Sullivan MJL, Bishop S, Pivik J. The pain catastrophizing scale: development and validation. Psychol Assess, 1995, 7: 524-532
