# Supplementary material for: The association between pain catastrophizing, physical function and pain in a cohort of patients undergoing knee arthroplasty
Source: BMC Musculoskelet Disord. 2019 Sep 12;20:421. doi: 10.1186/s12891-019-2787-6 (PMC6739909; doi:10.1186/s12891-019-2787-6)
Supplement: Supplementary file 2 — Additional analysis. Analysis with PCS cut-off score at 21 and 30. (DOCX 16 kb) [file 12891_2019_2787_MOESM2_ESM.docx]

**Additional analysis**

Analysis with PCS cut-off scores at 21 and 30

Table 2. Association between preoperative pain catastrophizing score and change in Oxford Knee Score (OKS) from preoperatively to 12 months postoperatively

|  | OKS total score | | |
| --- | --- | --- | --- |
| Variable | Change in OKS * | (95% CI) | P value |
| TKA (n=419)  PCS ≤20  PCS 21-30  PCS >30  UKA (n=162)  PCS ≤20  PCS 21-30  PCS >30 | ref  1.4  3.8  ref  3.6  3.4 | (-0.8 - 3.6)  (1.2 - 6.4)  (0.3 - 7.6)  (-0.7 – 7.5) | 0.22  0.004  0.04  0.105 |

*Adjusted for sex, age, BMI, alcohol, smoking, operated bilateral§ and co-morbidity.

§ Patients having two knee arthroplasties within 12 months.

Table 3: Association between preoperative pain catastrophizing score and pain measured 12 months after knee arthroplasty

|  | Moderate/severe pain  vs mild/very mild/no pain | | |
| --- | --- | --- | --- |
| Variable | Odds ratio* | (95% CI) | P-value |
| PCS≤20  PCS 21-30  PCS >30 | ref  2.3  2.3 | (1.3 – 3.8)  (1.3 – 4.2) | 0.002  0.006 |

*Adjusted for sex, age, BMI, alcohol, smoking, operated bilateral§ and co-morbidity.

§ Patients having two knee arthroplasties within 12 months.

Table 5. Differences in Oxford Knee Score (OKS) and SF-36 (PF) for patients with low and high preoperative Pain Catastrophizing Score (PCS)

|  | Low (n=391) vs high (n=91) PCS | | | | | |
| --- | --- | --- | --- | --- | --- | --- |
| Time | OKS | 95% CI | P value | SF36 (PF) | 95% CI | P value |
| Preoperative  4 months  12 months | 7.5  4.3  3.9 | (6.2 – 8.9)  (2.4 – 6.3)  (1.8 - 6.1) | 0.000  0.000  0.000 | 15.9  6.0  7.0 | (11.5 – 20.3)  (3.3 – 13.9)  (2.3 – 14.0) | 0.000  0.001  0.006 |
